# Harm Reduction as “Continuum Care” in Alcohol Abuse Disorder

**DOI:** 10.3390/ijerph121114828

**Published:** 2015-11-19

**Authors:** Icro Maremmani, Mauro Cibin, Pier Paolo Pani, Alessandro Rossi, Giuseppe Turchetti

**Affiliations:** 1 Vincent P. Dole Dual Disorders Unit, Department of Neurosciences, Santa Chiara University Hospital, University of Pisa, Pisa 56126, Italy; 2 Association for the Application of Neuroscientific Knowledge to Social Aims, AU-CNS, Pietrasanta, Lucca 55045, Italy; 3 G. De Lisio Institute of Behavioral Sciences, Pisa 56126, Italy; 4 Mental Health and Addictive Behaviors Department, Local Health Authority, Venice 30010, Italy; E-Mail: m.cibin@libero.it; 5 Social and Health Services, Health District 8 (Local Health Authority), Cagliari 09121, Italy; E-Mail: pallolo@tin.it; 6 Italian Society of General Practitioners, Firenze 50142, Italy; E-Mail: rossi.alessandro@simg.it; 7 Institute of Management, ©Scuola Superiore Sant’Anna, Pisa 56126, Italy; E-Mail: giuseppe.turchetti@sssup.it

**Keywords:** alcohol dependence, alcoholism, anti-craving medications, compliance with treatment, comprehensive treatment, detoxification, economic costs, harm reduction strategy, opioid receptors, public health, reduction in alcohol consumption, stigma

## Abstract

Alcohol abuse is one of the most important risk factors for health and is a major cause of death and morbidity. Despite this, only about one-tenth of individuals with alcohol abuse disorders receive therapeutic intervention and specific rehabilitation. Among the various dichotomies that limit an effective approach to the problem of alcohol use disorder treatment, one of the most prominent is integrated treatment *versus* harm reduction. For years, these two divergent strategies have been considered to be opposite poles of different philosophies of intervention. One is bound to the search for methods that aim to lead the subject to complete abstinence; the other prioritizes a progressive decline in substance use, with maximum reduction in the damage that is correlated with curtailing that use. Reduction of alcohol intake does not require any particular setting, but does require close collaboration between the general practitioner, specialized services for addiction, alcohology services and psychiatry. In patients who reach that target, significant savings in terms of health and social costs can be achieved. Harm reduction is a desirable target, even from an economic point of view. At the present state of neuroscientific knowledge, it is possible to go one step further in the logic that led to the integration of psychosocial and pharmacological approaches, by attempting to remove the shadows of social judgment that, at present, are aiming for a course of treatment that is directed towards absolute abstention.

## 1. Introduction

Alcohol abuse is one of the most important risk factors for health and is a major cause of death and morbidity. Despite this, in Italy only about one-tenth of individuals with alcohol use disorders (AUD) receive therapeutic intervention and specific rehabilitation. One evident handicap is thus the currently very limited access to treatment, which is usually dependent on the seriously inadequate availability of first-line interventions. Once the patient has “reached” treatment, the problem is posed of whether or not the treatment itself is adequate—A consideration that is correlated with the dropout rate.

In Italy, 454 services or working groups on alcohol dependence have fewer than 70,000 patients, with a mean age of 45.9 years, a figure which falls to 43.9 if only new entries are considered [1]. Most of the increase in patients over the years is due to patients who remain in long-term treatment, while the number of new patients has stayed relatively unchanged over time. The overall picture is now that of a heterogeneous system of intervention between different geographic areas, in which it is difficult to recruit new patients, in particular, younger individuals with a short-lived history of AUD. Many services tend to maintain the current patient load and find it difficult to network with other services, especially self-help groups.

A recent overview [2] found a 3.4% prevalence of AUD in the European population between 18 and 64. In Italy the population between 18 and 64 is about 37,600,000, which translates into an estimated 1,300,000 people in Italy suffering from AUD. It is estimated that at least 10 times more individuals need intervention than those actually receiving it. The current situation is correlated with several heterogeneous factors:

- “Cultural” factors, notably stigmatization,
- Factors related to the organization of services,
- Factors related to the setting up of treatment programs.

Concentrating now on cultural factors, resistance to entry into treatment is related to personal and social stigmatization: It is commonplace to view an alcoholic as a person who cannot be accepted and is severely marginalized and incurable. There is also the widespread perception that treatment centers for dependency are receptacles of marginalization and delinquency. The concept of alcohol use disorder as an “accident” that could happen to anyone as a result of a risky lifestyle and/or traumatic events undoubtedly helps to overcome such stigma, as does the awareness that the condition can be addressed and cared for with professional and scientifically validated tools. It is more difficult to deal with the stigma attached to treatment centers: These services are often located in areas which, both from geographical and relational points of view, are degraded. In addition, it should be highlighted that patients with AUD can only rarely exercise their right to the choice of care, considering the territorial distribution of centers for addiction and psychiatric services: The patient can only take into consideration the public service where he/she is a resident. It seems that cultural issues and factors related to the organization of services act synergistically to keep potential patients at a distance.

Among the organizational factors that make it difficult for patients to get access to services, the following should be considered:

- Distribution of services and availability of treatment, which is damaged by marked heterogeneity between regions, especially between northern and southern regions within Italy;
- Organization of services, which is dissimilar both at first contact (e.g., waiting times, fees) and the overall setting of treatment programs. Specific pharmacological treatments are definitely underutilized, as are self-help groups and residential programs.

For the purposes of access to treatment, however, the greatest shortcoming is the scarcity or, in many cases, total lack of first-line interventions. These last, by definition, are the foundation of the effective management of any problem of epidemiological importance comparable with that set by alcohol use disorders.

In terms of first-line intervention, the roles both of general practitioner and of specialists involved in the treatment of AUD (gastroenterologists, psychiatrists, neurologists) are fundamental. Current diagnostic and therapeutic tools can be used to propose effective interventions that are compatible with the context that comprises both the general practitioner and specialists—Interventions that can be carried out individually or through consultation with alcohol abuse services.

Other factors that can influence access to and facilitate the continuation of treatment are the setting of the program, in particular, the relationship with the patient. This aspect is fundamental in order for the intervention to be effective and to reduce the risk of early dropout. As for any therapeutic intervention, that is of particular importance in the treatment of problems arising from difficulty in motivational and decisional processes [3], as well as in a maladaptive use of memory [4,5]. Building a relationship, encouraging changes, supporting self-efficacy are all essential elements of a motivational approach, with the aim of defining, interactively with patients, the objectives and course of treatment.

Abstinence from alcohol use and other psychoactive substances would seem to be the most desirable goal, but not all subjects have the motivation or personal resources to reach this endpoint. In some cases it is possible to define an objective of reduction, which will at least keep the dangerous effects of alcohol to a minimum, while reducing the risks of dropout. In the words of Alan Marlatt: “If a client is ambivalent toward or resistant to changes, then harm reduction (…) gives us an opportunity to build a relationship and help our client make steps in the right direction (…). Reduction therapy means meeting the clients where they really are” [6].

## 2. The Need to Overcome the Dichotomy “Harm Reduction” *vs*. “Comprehensive Treatment”, Plus the Advantages of a Philosophy of Intervention that Refrains from Aiming at Immediate Disengagement from Alcohol

Among the various dichotomies that limit an effective approach to the problem of addiction, one of the most prominent is “integrated treatment *versus* harm reduction”. For years, these two strategies have been considered opposite poles of different philosophies of intervention. One is bound to the search for methods that lead the subject to complete abstinence, the other assigns top priority to a fall in substance use, with maximum reduction in the damage done correlated with the extent of the fall in use [7].

Supporters of harm reduction argue that this approach is desirable in any case, because it promotes each patient’s opportunity to improve his/her health while reducing the risk of practices of abuse. Critics argue that this approach is useless, as it does not intervene in the pathophysiology of the disease, maintaining the positive reinforcement of the intake of the drug and course of disease [8]. If, however, these considerations are questionable for drugs, they cannot be applied to alcoholism. In some cases the introduction of pharmacotherapy can facilitate the integration of these two different approaches to the problem [9].

AUD remains an underdiagnosed and undertreated condition. The problem in treating subjects with at-risk drinking, but who are not yet severely alcohol-dependent, is often the individual will of the subject, who does not want to immediately stop drinking despite realizing that a reduction in alcohol consumption is desirable.

The therapeutic agents used up to now have focused on the pharmacologically assisted detoxification of AUD patients and long-term psychosocial treatment of detoxified subjects. Alternatively the use of drugs to prevent or delay relapse into alcohol abuse in detoxified subjects have been used. To date, no drug has been proposed to facilitate a reduction in drinking. A reduction of alcohol intake in patients with alcohol use disorders can be considered an intermediate objective towards complete abstention. This is particularly true in severe AUD patients at risk of physical and psychological complications (reduction of risk of disease progression and/or damage).

The process of acceptance of harm reduction in alcoholism begins with the following findings: Alcohol consumption, when high (>1 unit per day for women and >2 units per day for men), is one of the most important risk factors for disease; the risk of death increases with alcohol intake in an exponential manner; at-risk drinking has negative consequences on the individual’s socioenvironmental condition (decreased productivity, disruption of meaningful relationships, violent and criminal behavior in both family and social environments, increase in injuries), with an exponential trend that is related to alcohol intake [10]. A progressive increase in alcohol consumption is the greatest risk factor for alcohol dependence, which, in its turn, is responsible for most of the psychophysical complications and social issues linked with drinking. Reducing the intake of alcohol therefore means reducing the risk of developing addiction, too [11].

Reducing alcohol consumption has an immediate positive impact on the health of the at-risk drinker. Immediate improvement is seen in sleep disorders, mood abnormalities, problems related to poor nutrition and blood pressure, all of which are strongly influenced by heavy drinking. It also lowers the risk of cirrhosis, cancer, cardiovascular complications, osteoporosis and pancreatitis, with the added advantage of a decrease in costs associated with the physical and mental complications of alcoholism. In addition, subjects who greatly reduce the use of alcohol, and those who completely interrupt their consumption, generally show the same benefits in terms of social adaptation [12,13,14].

Thus reducing alcohol intake without achieving complete abstention does not mean giving up the aim of treating the disease. It is possible to consider this type of intervention as an intermediate resource, which can lead over time to complete abstention from alcohol. Many patients who have agreed to reduce their drinking, initially supposing that this was their goal, changed their goal over time to achieving complete abstention [15].

The availability of opioids antagonists and anti-reward and anti-dysphoric drugs facilitates harm reduction in alcoholism especially if it is accompanied by psychosocial support. In this way it is possible to offer a valid integration with treatments oriented towards complete abstention, according to the following principles:

- Many patients prefer not to completely abstain from alcohol, even if they are aware of the risks;
- Reduction of alcohol intake can be thought of as an additional low-threshold treatment that is non-stigmatizing and stays flexible;
- The results of interventions aimed at alcohol reduction can be as successful as the immediate interruption of drinking;
- Reduction of alcohol intake is guided by an appropriate strategy in many guidelines for the treatment of alcoholism (EMA, NIAAA, NICE);
- A reduction in alcohol intake does not require any particular setting, but it does require collaboration between the general practitioner, specialized services for addiction, alcohology services and psychiatry.

## 3. The Role of the General Practitioner Who Keeps His/Her Focus on Addiction

Alcohol is one of the key determinants of human health. The strategies that national healthcare services put in place to solve alcohol-related problems inevitably tend to interfere with each another. With a specific focus on the general practitioner, the most straightforward approach will be to pose a few simple questions:

- Is it useful and necessary to extend current types of health intervention to alcohol?
- Should intervention be extended to primary care settings?
- What types of intervention should be considered within a primary care setting?
- Is the general practitioner adequately trained to intervene in alcohol addiction?
- What obstacles and difficulties should be taken into consideration?

### 3.1. Is It Useful and Necessary to Extend Current Types of Health Intervention to AUD?

Should intervention be extended to primary care settings? The general practitioner has the role of evaluating the patient’s lifestyle and approaches that are correlated with alcohol-related problems or disease, however they emerge. As for the screening and evaluation of interventions for problems related to the use of alcohol (as with tobacco and drugs), any type of intervention is realistically feasible, provided that there are clear objectives and limits. In the setting of general medicine, the status of alcohol-related problems covers a range of heterogeneous conditions. The situation is different in specialized centers, where patients present at an advanced stage of dependence and/or confirmed polyabuse. Even in these circumstances, the general practitioner has a key role to play. On the other hand, at a European level, the importance of prevention and early detection by the general practitioner is stressed, at least for target individuals often called “problem drinkers”, *i.e.*, those who have not yet been affected by addiction but are unwilling to reduce their drinking, even when that is recommended by their physician.

### 3.2. What Types of Intervention should be Considered within a Primary Care Setting?

Unquestionably, there are two types: Early detection and brief intervention. In some cases, pharmacological intervention can be employed. Concerning early detection, it is our belief that a periodic structured interview is not a real option for all patients. It is thus preferable to consider specific situations, previously recognized through individual case findings or in groups of individuals at particularly high risk. The general practitioner must therefore resort to the scrupulous recording of medical history of alcohol consumption and of any events, symptoms, or signs that would be useful in identifying subjects who warrant a more thorough assessment. In this connection, the administration of tests may be useful, such as the AUDIT-C, which, according to WHO, is the most reliable test in primary care settings. It should, however, be noted that an informal and open interview seems to provide sensitivity, specificity and predictive values that substantially overlap structured questionnaires [16,17].

In subjects in whom a problem has emerged, and in those with a positive AUDIT score, brief intervention is desirable. Such intervention has been shown to be significantly effective by many studies (especially those in the setting of general medicine) in terms of reducing alcohol consumption. Brief intervention is sustainable in terms of time and is workable in terms of the educational and communicative competence of the general practitioner. Lastly, a careful review of new treatment methods relying on pharmacological therapy for alcoholism, and the availability of easy-to-manage drugs, allows the general practitioner to provide selected cases with medical therapy.

### 3.3. Is the General Practitioner Adequately Trained to Intervene in AUD?

The answer to this question is potentially complex and protracted. For the sake of brevity, we can consider the kind of general training that is given at medical school; the many constraints to be found in current practice; the organizational barriers; and the lack of defined pathways for clinical care. All these often lead the general practitioner to underestimate this type of problem in his/her patients.

### 3.4. What Obstacles and Difficulties Should be Taken into Consideration?

To some extent the answer to this question overlaps with the previous one. The summary of the explanations given below was taken from a survey carried out by the Italian Society of General Practitioners on prior training activities. The motivations provided in this light were: (i) lack of time; (ii) fear of conflicts with the patient or that might promote conflicts within the family or the couple; (iii) perception of limitation or lack of effectiveness of treatment; (iv) the belief that patients with this type of problem do not show any, or only a poor response to treatment; and (v) inadequate knowledge of counseling techniques and brief intervention.

In conclusion, a simple but effective “package” of therapeutic tools, used in the setting of general medicine in collaboration with the specialist, could include: (i) motivational counseling reinforced by relapse prevention; (ii) pharmacotherapy, and (iii) referral to specialized services and self-help groups.

## 4. AUD Patients and the Network of Territorial Services

There are some areas that, given the complexity of the healthcare and social factors involved, are more amenable to an integrated approach in which the specialization and fragmentation of interventions can result in damage. This is especially true where specialists tend to focus on their obligations rather than communicating effectively [18]. Alcohol dependence treatment requires close connections between psychosocial interventions and pharmacological therapy, continuity of care and case management. The integration and coordination of these different types of interventions, leads to rising standards of care and services, so offering to the patient undisputed benefits [19,20,21,22].

Apart from the wide-ranging positive outcomes that can be achieved, there still remains the problem of integration between professionals, services and institutions [23,24,25,26]. In a national context, the case of alcoholism may be emblematic, as the sectorial approach affects the integration of interventions for alcohol dependence and those for dependence on other substances, even when they involve the same individual. In a National Health Service, optimal integration for interventions that aim to treat addiction may be best achieved by an orthogonal matrix system where each addiction unit is placed in one health district and in one department. A health district implies a horizontal structure that ensures the integration of the health and social services in a specific urban or rural area. A department implies a vertical service line within the organizational structure that ensures the scientific-technical quality of interventions. In this type of healthcare organizational model, services for addiction can be based on a “hub and spoke” pattern, which concentrates the general functions of operational planning, coordination and clinical services in a hub. The more specific “nodes” and “points” should function as “spokes”: Their activity is closely integrated with the hubs, and distributed throughout the territory. They take the form of structures and operational realities that can more easily fulfill requests for assistance (operational territorial units: Those treating problems such as alcoholism and smoking; general practitioner outpatient departments; mental health centers, family planning clinics, social services, and so on). Such a scheme has the aim of ensuring a uniform system, and makes timely use of technical and professional skills and resources anywhere within the network. In this way transfers of clients to specific situations and time phases can be limited, on the basis of the overall therapeutic and rehabilitative course. The activation of this type of system could start with pilot projects, similar to those implemented in the UK [27], with the participation of services for addiction and other key healthcare and social structures.

Further interventions could aim to encourage the development of skills in achieving defined levels of integration. In fact, current systems of payment for services usually fail to take into account the value of integration and coordination, and do not provide incentives to promoting dialog between the different levels of care management [28,29]. The inclusion of care continuity among the basic levels of care should also be considered in terms of its economic value, especially in view of the fact that the current evidence is limited to the cost-effectiveness of selected integrated care approaches, while the quality of evidence is still low [30].

In any case, the establishment of an appropriate level of assistance to ensure the integration and continuity of care implies the adoption of legislative and regulatory choices that resolve critical issues. These last are linked with general forms of integration, both horizontal (linking institutions and services, and, similarly, healthcare and social operators) and vertical (linking key structures, services and operators that define the functions of basic, specialist and hospital care). All this will help to integrate the different areas of expertise, organization, performance and forms of financing [31,32].

## 5. The Care Continuum and Economic Prospects for Setting up a New Paradigm for the Therapeutic Management of AUD Patients

AUD is not only a serious public health problem; it is also a major cause of expense, in terms both of healthcare and social resources. In Europe, it is estimated that social expenses related to alcohol amount to about €155.8 billion. In Italy alone they are estimated to be €22 billion [33]. Undoubtedly, the magnitude of social costs, which include direct healthcare and non-healthcare costs, indirect costs and intangible costs associated with alcoholism, must be kept in mind when developing strategies to adequately address the phenomenon of alcoholism and its direct implications.

Spreading awareness of the importance of the overall health and economic burden caused by alcoholism is, in fact, the first step towards an effective management of the problem. Long-term strategies are needed, to be supported by an adequate, recurrent allocation of funds capable of generating effective preventive measures and profound cultural change. On one hand, this would help to lower the number of individuals who become alcoholics, and, on the other, help those who approach the treatment centers for alcohol addiction in a society that has overcome much of the stigmatization of alcohol dependence. These strategies will make it possible to achieve the best results from an economic point of view.

What is, in any case, clear is that the strategies that will be able to provide significant results in the short to medium term should be achieved through the optimization of the diagnostic-therapeutic path that is provided for alcohol-dependent patients. In fact, by redirecting the care continuum toward a better, more systematic coordination and organization of all the actors involved in the process, so maximizing skills and intervention strategies, health indicators can be improved and the economic burden on society due to alcoholism can be lightened.

What, then, is the best model for the treatment of alcohol dependence? Without question, it is the model that makes it possible to achieve abstention, the “best of the best”—The absolute best, when reviewing the health, economic and social viewpoints. Whether in medicine or economics, it should be recognized that the pursuit of the “absolute best” has to move through a longer or shorter sequence of “relative bests”, as that is the only way to achieve the optimal results achievable in the given conditions. Thus, the question “What is the best model for the treatment of alcohol dependence?” has to be reinterpreted as “What is the best available model for treating the individual patient now, in the current phase of his/her disease, fully taking into account the specific clinical, personal, family, and environmental conditions?”. What is the realistic target, allowing for these particular constraints? In this way, the search for the absolute best becomes a more empirical search for the relative best—In this case, for example, moving from achieving total abstention to achieving harm reduction. By hitting that target and stabilizing the situation, the patient enters a different management phase, where the situation calls for another target to be set by moving on to another relative best. Viewed from an economic standpoint, therefore, the search to reduce the worst possible accumulation of harm is not a renunciation (nor an abdication, or admission of impotence) of aiming for a “best of the best” objective—In this case, abstention and total recovery of the patient—But the pursuit of an intermediate target, a relative best, by systematically moving forward in the direction of the absolute best. It must, in any case, be kept in mind that for some patients the relative best may be the only result to strive for in the medium to long term. While this may not be completely satisfying, from an economic standpoint it is certainly preferable to be satisfied with reducing harm and achieving an “acceptable” target, rather than failing completely in the pursuit of a more desirable “best of the best” that is simply not achievable.

What is the economic impact, and the social cost, of a therapeutic strategy for the alcoholic patient, when the target is harm reduction? From a clinical point of view, the availability of instruments that allow differentiation of the therapeutic strategy, depending on the stage of the disease, and on family and social conditions, is clearly desirable. In addition, the greater variety of targets and therapeutic strategies opened up by making harm reduction the target of the treatment would make it possible, even from an economic point of view, to reduce the burden of the disease on society. In fact, by definition, an intermediate suboptimal target, a relative best, is more likely to be achieved rather than the ultimate optimal goal of absolute elimination of alcohol-related harm. Thus, in patients who hit the desired target, significant savings in terms of health and social costs will actually be achieved. Thus harm reduction turns out to be a desirable target, even when an economic standpoint is adopted.

A greater personalization of the targets, is the strategy most likely to further reduce the burden of alcoholism on society, even when the overall assessment includes the economic criterion. In particular, the therapeutic approach that is consequent on a situation of care continuum, will allow the achievement of a series of intermediate targets—A dynamic sequence of relative optima—That will emerge more clearly when the situation is surveyed from a clinical vantage point.

## 6. Towards the Future—The Need for a Care Continuum Even in AUD Patients

Increased interest in reducing at-risk drinking—A strategy made possible by new therapeutic options—May, in the near future, open the way forward to an innovative therapeutic organization for alcohol use disorder. As for other medical specialties, assistance will be organized by distinguishing between the levels of intervention [8,34,35,36]. There is, in fact, no scientific evidence of better results in the case of non-adoption of the general principles of treatment for chronic diseases (criterion J). Patients with alcohol use disorder should be treated as normally as possible, without resorting to the established schemes that tend to be based on rigid and stigmatizing rules (criterion K) [37,38,39].

**Figure 1:**
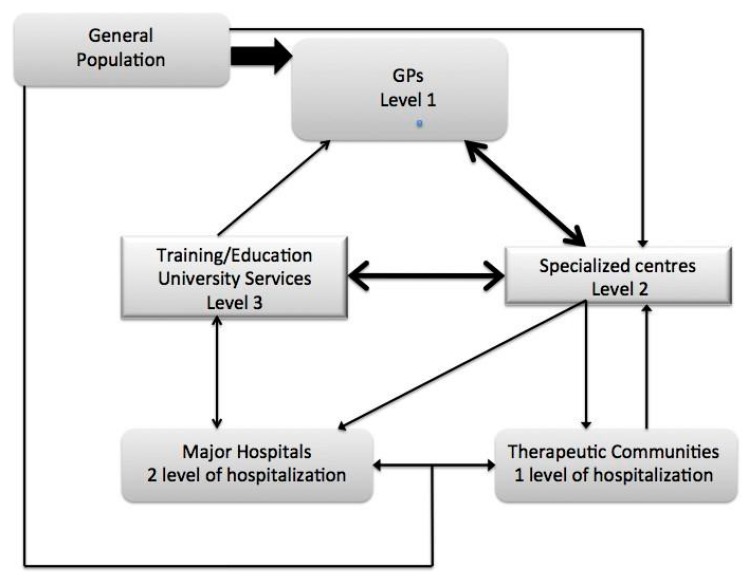
New organization in treating AUD patients.

Level 1 (the first level of intervention) is the responsibility of the general practitioner; level 2 (the second level of intervention) includes specialized services dedicated to alcoholism, drug abuse and dual diagnoses, in addition to problem drinking in psychiatric patients. In this way it becomes possible to intervene in a selective manner on the successive phases of alcohol use disorder, from onset to psychophysical decompensation, and even the second level of severity). A patient could, therefore, be treated at the outpatient clinic of his/her general practitioner’s or psychiatrist’s office, without needing to move outside the National Health Service, and later go back to being under their observation after specialist intervention for increasing severity. Level 3 (the third level of intervention) comprises all the services at university clinics that are specifically dedicated to non-responders or particularly complex cases. The general practitioner will work as an intermediary between the general population and specialized centers. Specialized services will operate as outpatient facilities and as shelter facilities through agreements with therapeutic communities (first level of admission) and, in person, at hospitals (second level of admission). The University will operate as the third level of assistance through specific outpatient services and hospitalization for treatment-resistant patients; knowledge will be transmitted through teaching (medical degree, nursing, psychological and sociological disciplines), specialization, research and postgraduate teaching (master level 1 and 2, CME in medicine) (see Figure 1). This model, widely used for all chronic diseases, is defined as “shared care” or a “mixed care model”. Only this model can adequately treat alcohol use disorder by minimizing negative interference that arises out of the rigidity of treatments and forms of stigma that are currently experienced [40].

In an integrated vision of the disorder, work is needed on a cultural level to reinforce the complementary nature of psychosocial and pharmacological interventions that are not specifically dedicated to promoting or maintaining a state of immediate abstinence from alcohol. In addition, this is especially true in order to offset the marginalization, often largely hidden, to which patients with alcohol use disorder are usually subjected when they “do not want” or “cannot at the moment” stop drinking completely. Integration, without any doubt, will be the cornerstone of this activity, and will form the core of a new philosophy of integration between therapeutic approaches. Patients must be integrated into the civil society within which they have become dependent. In this situation, in order for them to be publicly recognized as having been cured, they must actually be cured, so creating a genuine and concrete path of recovery. That path should extend from the reduction of drinking that exposes them to severe risks through to complete abstention.

Pharmacological intervention in itself, acting as the agonist or antagonist of the opioid system, even if extremely innovative, is not enough to bring about a new philosophy of addiction treatment. Those who cure depression know that drugs which bring about a faster return of the patient to employment, limit the impact of the care pathway on social life and allow patients to be cured within their social context.

For alcoholism, such reasoning has struggled to emerge when the drug in question allows “only” a decrease in at-risk behavior [12,41,42,43].

At the present state of neuroscientific knowledge, one step further can be made by extending the logic that led to the integration of psychosocial and pharmacological approaches, so as to remove the stigma of social judgment aiming for a course of treatment directed towards absolute abstention. In fact, when abstention is not considered the ideal therapeutic target for a given patient because he/she is unwilling to accept it—A reaction that amounts to a *de facto* barrier to treatment—New therapeutic modalities should be identified, together with integrated approaches that motivate patients to move forward along a path of individualized treatment.

This allows for taking care of individuals who would otherwise never be prepared to enter into a rigid path of care, even when they are on the verge of losing their role in their family and in society. Accompanying these patients through the reduction of alcohol intake toward the final target of complete abstention will be a historical evolution, which can only be achieved by investing in a network of comprehensive services that are integrated within healthcare and social structures.

## 7. Conclusions

In the treatment of AUD patients, at the present state of neuroscientific knowledge, it is possible to go one step further in the logic that led to the integration of psychosocial and pharmacological approaches, by attempting to remove the shadows of social judgment that, at present, are aiming for a course of treatment that is directed towards absolute abstention.
